# Prognosis of adenoid cystic carcinoma in head and neck region treated with different regimens—A single‐centre study

**DOI:** 10.1002/cam4.5065

**Published:** 2022-08-07

**Authors:** Yutian Cheng, Le Xu, Zhanwei Chen, Haiwei Wu, Huwei Zou, Tianqi Zhang, Guijun Liu, Zhenxing Liu, Changwei Yin, Li Ma, Shizhou Zhang, Wengang Li, Shengyun Huang, Dongsheng Zhang

**Affiliations:** ^1^ Department of Oral and Maxillofacial Surgery Shandong Provincial Hospital Affiliated to Shandong First Medical University Jinan Shandong China

**Keywords:** ^125^I seed radiotherapy, adenoid cystic carcinoma, oral and maxillofacial region, perineural invasion

## Abstract

**Background:**

No study has evaluated the impact of regimen on recurrence, metastasis and survival in patients with adenoid cystic carcinoma (ACC). The present study aimed to compare the efficacy of radioactive seed implantation and other regimens in treating ACC, so as to investigate the clinical applicability of radioactive seed implantation and determine the indications for this regimen.

**Methods:**

A total of 188 patients with ACC in oromaxillofacial region were allocated to four groups according to the treatment regimen: group 1 was treated with a combination of surgery and ^125^I seed therapy, group 2 with a combination of surgery and external radiotherapy, group 3 with surgery, whereas group 4 was untreated. The Kaplan–Meier method was used to assess the survival rates, and the Cox regression analyses were used to identify the associated prognostic factors.

**Results:**

The overall survival rates of 188 patients and groups 1, 2, 3 and 4 were 85.7%, 75%, 68.2% and 37.5%, respectively. Cox regression analysis revealed that age, T stage, N stage and regimen were independent prognostic factors of survival. Amongst patients with primary ACC, the efficacy of radioactive seed implantation was higher in those with perineural invasion than in those without.

**Conclusion:**

Patient age, T stage, N stage and regimen are independent prognostic factors of survival in patients with ACC. Patients treated with surgery combined with postoperative ^125^I seed radiotherapy have a higher overall survival rate, and those with perineural invasion are more suitable for radioactive seed implantation therapy.

## Graphical abstract

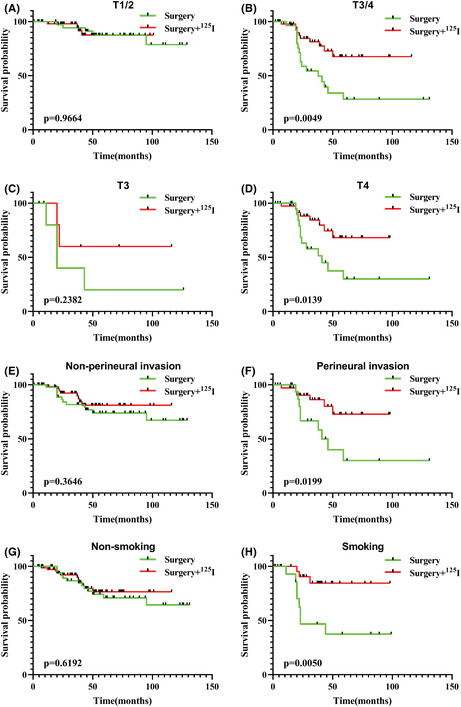

## 1 INTRODUCTION

Adenoid cystic carcinoma (ACC) is a malignant tumour of glandular origin and is prone to recurrence and metastasis, accounting for 30.7% of salivary gland malignancies.^1^ The 5‐ and 10‐year relative overall survival rates after diagnosis are 78% and 65%, respectively.^2^ ACCs grow slowly compared to other tumours but are prone to nerve invasion, which causes local pain, facial paralysis and other symptoms.^3^, ^4^ Failure to initiate timely treatment may cause tumour growth and compression of adjacent vital organs such as the respiratory and digestive tracts, or distant metastasis, which may be life‐threatening.^5^ At present, the standard treatment is a combination of radiotherapy and surgical removal of the primary tumour in order to reduce recurrence and improve survival.^6^, ^7^, ^8^ However, the role of postoperative radiotherapy in the adjuvant treatment of ACC remains controversial, with Garden et al.^9^ and Al‐Mamgani et al.^10^ reporting excellent control rates with postoperative radiotherapy, in contrast to Li et al.^11^ Chen et al.^12^ and Iseli et al.^2^ reporting no significant benefit of postoperative radiotherapy. ^125^I seed brachytherapy has high conformality and can achieve low‐dose, low‐damage yet high‐efficiency antitumour effects by adjusting the position of the radioactive seeds for a proper distribution of the prescribed dose in the overlapping radiation fields.^13^, ^14^, ^15^ One study investigated the local area control rate, tumour‐free survival rate and overall survival rate in patients with ACC who were treated with surgery combined with ^125^I seed therapy and reported good clinical outcomes.^16^ However, no study has evaluated the impact of regimen on recurrence, metastasis and survival in patients with ACC. The present study aimed to analyse the differences between different regimens in terms of the recurrence, metastasis and overall survival rates amongst patients with ACC, so as to provide a basis for evaluating the applicability of radioactive seed implantation in the treatment of ACC of the oral and maxillofacial region and identifying the indications for this regimen.

## 2 MATERIALS AND METHODS

### 2.1 Study subjects

A total of 188 patients with ACC of the head and neck who were diagnosed and treated in the Department of Oral and Maxillofacial Surgery of the Shandong Provincial Hospital affiliated to Shandong First Medical University between January 2010 and December 2020 were retrospectively analysed. All cases were confirmed by pathological examination; there was no history of radiotherapy, and no distant metastasis was found at the time of treatment. The diagnosis of ACC was based on pathology reports, and tumour staging was performed in compliance with the TNM staging system of the American Joint Committee on Cancer (8th edition). Demographic, epidemiological, clinical, pathological and treatment data were obtained from the patients' medical records, and follow‐up was conducted via evaluation of medical records and telephone interviews. The follow‐up time was defined as the time interval from the date of ACC diagnosis to the date of death or final follow‐up, which was used for calculating the overall survival rate. This study protocol was approved by the Biomedical Research Ethics Committee of Shandong Provincial Hospital (Approval number: SWYX: NO. 2021‐415). The need for informed consent was waived owing to the retrospective nature of the study.

### 2.2 Regimens

#### 2.2.1 Surgery

Surgical treatments were performed after the diagnosis of ACC was confirmed histologically, including partial resection of the primary tumour, local extended resection, combined radical surgery, selective neck dissection and therapeutic neck dissection.

#### 2.2.2 Radiotherapy and ^125^I‐seed therapy

^125^I is a synthetic isotope with a half‐life of 59.63 days and an effective radiation radius of 1.7 cm, releasing X‐rays of 27.4–31.4 keV and γ‐rays of 35.5 keV during decay.^17^ Model 6711 ^125^I seeds (HTA Co., Ltd.) were used in the present study, each with a length of 4.5 ± 0.3 mm, a diameter of 0.8 ± 0.03 mm and radioactivity of 0.6–0.7 mCi. The patients underwent intraoperative implantation of ^125^I seeds or secondary implantation of ^125^I seeds 4–6 weeks after surgery, with a dose of 60–80 Gy for patients with negative surgical margins and 80–120 Gy for patients with positive surgical margins.^16^ External radiotherapy was performed 4–6 weeks after surgery at a dose of ≥60 Gy for negative surgical margins and ≥66 Gy for positive surgical margins.^9^

### 2.3 Statistical analysis

Post‐treatment survival rates were calculated using the Kaplan–Meier method. Univariate variables with significant effects on survival were preliminarily identified and then further subjected to multivariate Cox regression analysis. *p*‐value of <0.05 was considered statistically significant. Statistical analyses were performed using SPSS 26 (IBM Corporation).

## 3 RESULTS

The study cohort comprised 188 patients (102 females and 86 males) aged 20–89 years, with a median age of 60 years. Forty‐four patients had died at the completion of follow‐up, with an overall survival rate of 76.6% and a median survival time of 39 months. The median tumour size was 2.5 ± 1.47 cm (range: 0.3–10 cm). Ninety‐eight patients underwent surgery, followed by ^125^I seed therapy (6–90 seeds implanted with a median number of 35.5 seeds), 16 underwent combined surgery and external radiotherapy, 66 underwent surgery only and 8 received no treatment, with survival rates of 85.7%, 75%, 68.2% and 37.5%, respectively. Of the 44 patients who died, 13.3% died from recurrence and 10.1% died from distant metastases.

Univariate Cox regression analyses were performed to identify potential prognostic factors of survival (Table 1). The results showed that age (*p* = 0.0016), regimen (*p* = 0.0059), T stage (*p* < 0.0001), N stage (*p* = 0.0257), bone invasion (*p* = 0.0002), smoking (*p* = 0.0293) and glandular site of onset (*p* = 0.018) were significant prognostic factors of survival in patients with ACC. Multivariate Cox regression analysis of these factors confirmed that age (*p* = 0.044), regimen (*p* = 0.013), T stage (*p* < 0.0001) and N stage (*p* = 0.029) were significant prognostic factors.

**TABLE 1:** COX regression analysis of the overall survival of ACC patients

Items	Data	Death	Alive	Univariate analysis	Multivariate analysis
Follow‐up time/months, median (range)	39 (0.8–131)				
Age/years, median(range)	20–89 (60)			0.0016**	0.044*
<60	94	14	80		
≥60	94	30	64		
Gender, *n* (%)				0.2658	
Male	86	22	64		
Female	102	22	80		
Treatment				0.0059**	0.013*
Surgery	66	21	45		
Surgery and radiotherapy	16	4	12		
Surgery and ^125^I brachytherapy	98	14	84		
Untreated	8	5	3		
T stage, *n* (%)				<0.0001****	<0.0001****
T1–T2	104	12	92		
T3–T4	84	32	52		
N stage, *n* (%)				0.0257*	0.029*
N0	174	38	136		
N1–N2	14	6	8		
Bone invasion				0.0002***	0.176
Yes	20	11	9		
No	168	33	135		
Nerve invasion				0.0669	
Yes	61	17	44		
No	127	27	100		
Smoker				0.0293*	0.19
Yes	47	15	32		
No	141	29	112		
Alcohol use				0.1525	
Yes	27	8	19		
No	161	36	125		
Surgical margins, *n* (%)				0.4491	
Positive	19	6	13		
Clear	169	38	131		
Salivary glands or other				0.018*	0.379
Salivary glands	182	40	142		
Other	6	4	2		

Abbreviation: ACC, adenoid cystic carcinoma.

* *p* < 0.05

** *p* < 0.01

*** *p* < 0.001

**** *p* < 0.0001.

**FIGURE 1:**
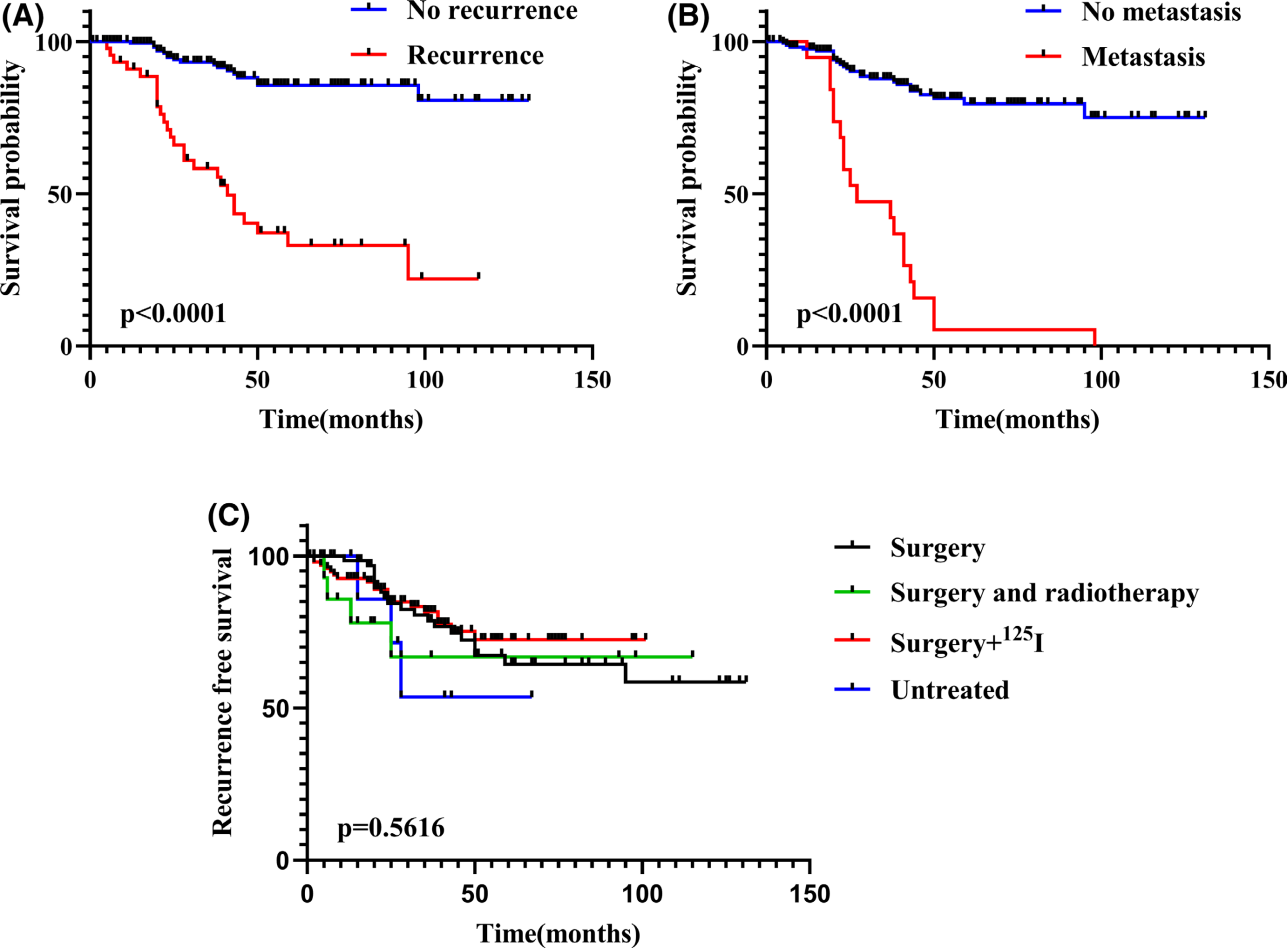
Clinical association of local recurrence (A) and distant metastasis (B) with the prognosis of patients with ACC. Recurrence‐free survival analysis of different therapies in ACC patients (C). ACC, adenoid cystic carcinoma.

Adenoid cystic carcinoma is prone to distant metastasis and recurrence. The Kaplan–Meier survival analysis revealed that local recurrence (*p* < 0.0001, Figure 1A) and distant metastasis (*p* < 0.0001, Figure 1B) were significantly associated with the prognosis of patients with ACC. Further analyses were conducted to identify which factors would influence distant metastasis and recurrence. The results indicated that 45 of the 188 patients experienced recurrence (Table 2). Univariate Cox regression analyses revealed that age (*p* = 0.0271), T stage (*p* < 0.0001), bone invasion (*p* = 0.0051), perineural invasion (*p* = 0.007) and glandular site of onset (*p* = 0.0045) were significant factors. However, there was no significant association between regimen and ACC recurrence (Table 2; Figure 1C). Further multivariate Cox regression analysis revealed that age (*p* = 0.003), T (*p* < 0.0001) and perineural invasion (*p* = 0.022) were significantly associated with recurrence.

**TABLE 2:** COX regression analysis of local recurrence in ACC patients

Items	Data	Recurrence	No recurrence	Univariate analysis	Multivariate analysis
Follow‐up time/months, median (range)	39 (0.8–131)				
Age/years, median (range)	20–89 (60)			0.0271*	0.003**
<60	94	19	75		
≥60	94	26	68		
Gender, *n* (%)				0.9864	
Male	86	19	67		
Female	102	26	76		
Treatment				0.4413	
Surgery	66	19	47		
Surgery and radiotherapy	16	4	12		
Surgery and ^125^I brachytherapy	98	19	79		
Untreated	8	3	5		
T stage, *n* (%)				<0.0001****	<0.0001****
T1–T2	104	12	92		
T3–T4	84	33	51		
N stage, *n* (%)				0.159	
N0	174	41	133		
N1–N2	14	4	10		
Bone invasion				0.0051**	0.478
Yes	20	9	11		
No	168	36	132		
Nerve invasion				0.007**	0.022*
Yes	61	20	41		
No	127	25	102		
Smoker				0.1606	
Yes	47	12	35		
No	141	33	108		
Alcohol use				0.4223	
Yes	27	6	21		
No	161	39	122		
Surgical margins, *n* (%)				0.3476	
Positive	19	7	12		
Clear	169	38	131		
Salivary glands or other				0.0045**	0.362
Salivary glands	182	41	141		
Other	6	4	2		

Abbreviation: ACC, adenoid cystic carcinoma.

* *p* < 0.05

** *p* < 0.01

**** *p* < 0.0001.

As shown in Tables 3, 19 of the 188 patients had metastases. Univariate Cox regression analyses revealed that age (*p* = 0.0207), N stage (*p* = 0.008), smoking (*p* = 0.0251) and alcohol consumption (*p* = 0.0485) were significant factors. Further multivariate Cox regression analysis revealed that only N stage (*p* = 0.014) was significantly associated with recurrence.

**TABLE 3:** COX regression analysis of distant metastasis in ACC patients

Items	Data	Metastasis	No Metastasis	Univariate analysis	Multivariate analysis
Follow‐up time/months, median (range)	39 (0.8–131)				
Age/years, median (range)	20–89 (60)			0.0207*	0.097
<60	94	6	88		
≥60	94	13	81		
Gender, *n* (%)				0.1531	
Male	86	11	75		
Female	102	8	94		
Treatment				0.1782	
Surgery	66	7	59		
Surgery and radiotherapy	16	2	14		
Surgery and ^125^I brachytherapy	98	8	90		
Untreated	8	2	6		
T stage, *n* (%)				0.2597	
T1–T2	104	11	93		
T3–T4	84	8	76		
N stage, *n* (%)				0.008**	0.014*
N0	174	15	159		
N1–N2	14	4	10		
Bone invasion				0.1721	
Yes	20	3	17		
No	168	16	152		
Nerve invasion				0.8644	
Yes	61	4	57		
No	127	15	112		
Smoker				0.0251*	0.087
Yes	47	8	39		
No	141	11	130		
Alcohol use				0.0485*	0.44
Yes	27	5	22		
No	161	14	147		
Surgical margins, *n* (%)				0.4928	
Positive	19	1	18		
Clear	169	18	151		
Salivary glands or other				0.4699	
Salivary glands	182	18	164		
Other	6	1	5		

Abbreviation: ACC, adenoid cystic carcinoma.

* *p* < 0.05

** *p* < 0.01.

**FIGURE 2:**
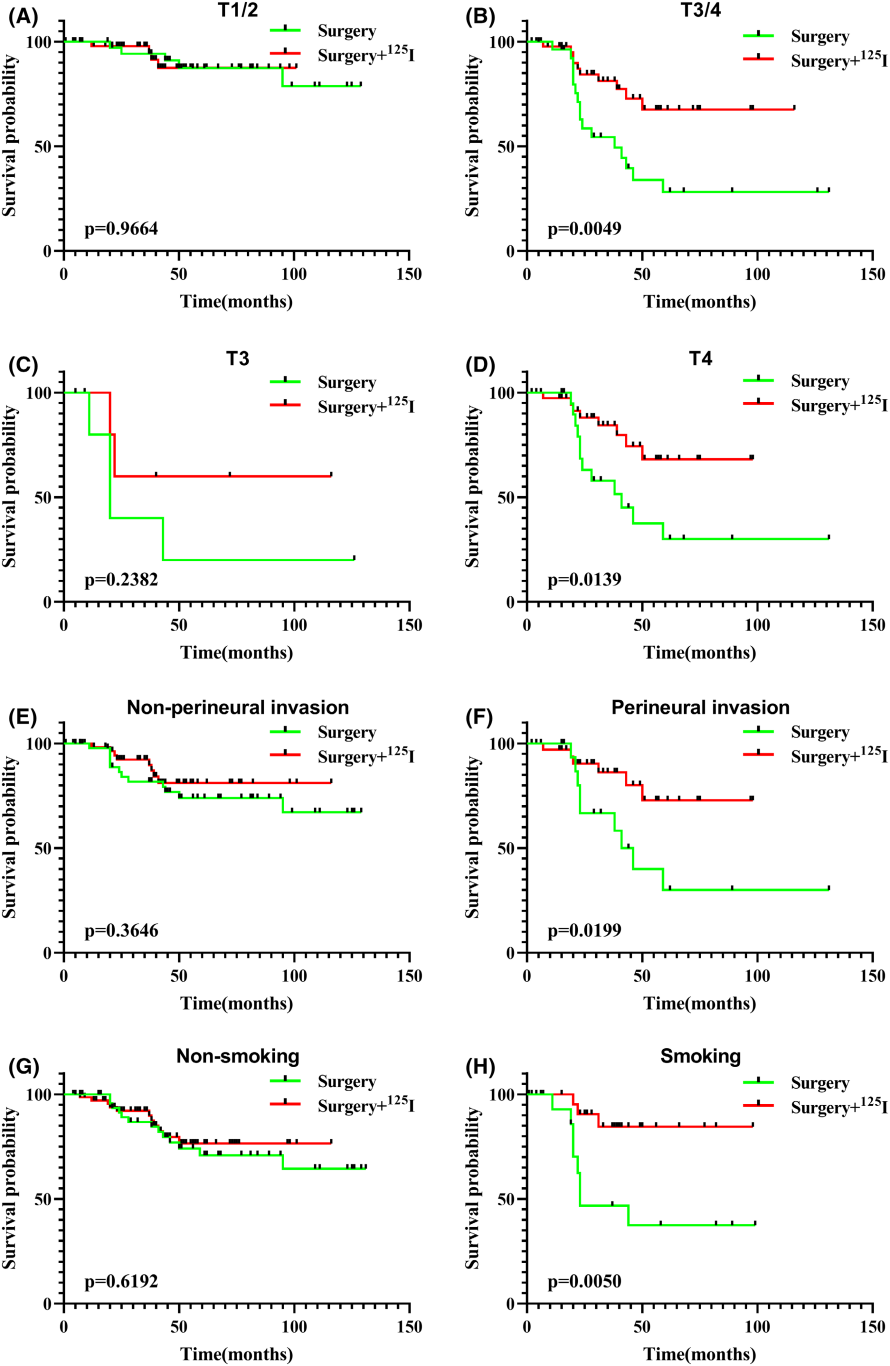
Survival analysis of ^125^I seed therapy in ACC patients with T1/2 stage (A), T3/4 stage (B), T3 stage (C), T4 stage tumours (D), and without (E) or with (F) perineural invasion, and non‐smoking (G) or smoking (H). ACC, adenoid cystic carcinoma.

Furthermore, the 188 patients were grouped by the aforementioned factors found to be significantly associated with ACC prognosis in order to explore their effects on the efficacy of ^125^I seed therapy. The results indicated that ^125^I seed therapy significantly improved the survival of ACC patients with T3/4 stage tumours (*p* = 0.0049, Figure 2B), but did not significantly affect survival in patients with early‐stage tumours (*p* = 0.9664, Figure 2A). Further comparative analyses revealed that the sensitivity of ^125^I seed therapy was lower in patients with T3 stage tumours (*p* = 0.2382, Figure 2C) than in patients with T4 stage tumours (*p* = 0.0139, Figure 2D). Some of the T4 stage patients developed perineural invasion (Figure 2E,F), leading to a higher therapeutic efficacy. Smoking (*p* = 0.0050, Figure 2H) also had a significant effect on the efficacy of ^125^I seed therapy compared with non‐smoking (*p* = 0.6192, Figure 2G).

## 4 DISCUSSION

Our findings indicated that age, regimen, T stage and N stage were the main influencing factors of survival, whilst smoking and gland type (gland or non‐gland type) were only slightly associated with survival, a finding similar to those reported by Westergaard‐Nielsen et al.^18^ and Coca‐Pelaz et al.^7^

The overall survival rate was 85.1% for patients aged <60 years and 68.1% for those aged ≥60 years. The lower survival rate of elderly patients was generally attributable to the following two factors: (1) as they generally suffer from life‐threatening systemic diseases, elderly patients often neglect small, slow‐growing maxillofacial masses, which results in a delay in treatments; and (2) elderly patients are unable to undergo aggressive treatments because of their systemic condition.^19^ The primary tumour site was another important influencing factor of survival; the reported risk of death from other primary tumours is higher than that from primary salivary gland tumours (*p* = 0.018).^16^ High T stage (*p* < 0.0001) and high N stage (*p* = 0.029) also affected overall survival in patients with ACC. Two additional factors, bone invasion (*p* = 0.0002) and smoking (*p* = 0.0293, univariate), were also associated with survival.^20^, ^21^

Age (*p* = 0.003), T stage (*p* < 0.0001) and perineural invasion (*p* = 0.022) were primary influencing factors of recurrence, whilst bone invasion and gland type (gland or non‐gland type) were only secondary factors. These observations can be explained as follows: (1) tumours in older patients are more inert and slow‐growing and often undetectable compared with those in younger patients; and (2) high T‐stage tumours and tumours with perineural invasion are often difficult to completely resect because of the larger tumour size, and consequently residual tumour foci continue to grow, ultimately leading to tumour recurrence.^4^

N stage (*p* = 0.014) was the primary influencing factor of distant metastasis, whilst age (*p* = 0.0207), smoking (*p* = 0.0251) and alcohol consumption (*p* = 0.0485) were only secondary influencing factors. Due to the slow growth of ACC,^19^ even if ACC metastasizes to the cervical lymph nodes, the metastases are likely undetectable on pathological examination due to their small size. That is, those lymph node metastases detected on pathological examination were either formed long ago or indicated an extremely strong propensity for metastasis, thereby suggesting a high likelihood of distant metastasis. Meanwhile, lymph node metastases may be detected during functional neck dissection in patients who do not present with clinically apparent lymph node metastases. The detected lymph node metastases, although small in size, may have been formed long ago without clinical signs, which may also lead to the eventual formation of distant metastases.

The survival rate is significantly higher in ACC patients receiving conventional treatments than in those who are untreated.^2^ Surgery is generally recommended for resectable ACC, but the histological characteristics of ACC and the complex anatomy of the maxillofacial region make it impossible to achieve complete resection by surgery alone, resulting in a poor prognosis.^9^ Adjuvant external radiotherapy is typically performed if there are adverse risk factors such as positive surgical margins, perineural and bone invasion, lymph node metastasis or advanced tumour staging.^12^ Surgery combined with postoperative radiotherapy can avoid the resection of nerves that are well known to be affected by the tumour during surgery.^22^ Iseli et al.^2^ reported that the survival rates associated with surgery combined with postoperative radiotherapy were higher than those associated with surgery or radiotherapy alone, and that the 10‐year local recurrence‐free survival rate with radiotherapy alone (0%) was significantly lower than that with surgery alone (41.8%) or with combined surgery and postoperative radiotherapy (43.5%), whilst there was no significant difference between the latter two. The above observations may be attributed to the fact that patients treated with radiotherapy alone typically receive palliative care owing to poor physical condition or high tumour malignancy (such as inoperable tumours, comorbidities, T4 stage tumours or large tumour size), and these factors lead to a low long‐term survival rate.^23^ In contrast, postoperative radiotherapy was not effective in improving the postoperative prognosis because external radiotherapy could not accurately locate the lesion area to achieve effective radioactive killing of the cancer cells, which suggests that ACC treatment requires more precise and efficient local irradiation.^12^ Further studies have demonstrated that postoperative radiotherapy does not improve 10‐year overall and disease‐free survival, but does improve local control rates and reduces tumour recurrence and metastasis. Therefore, radiotherapy alone is generally used only in the palliative care of patients with recurrence and distant metastases,^2^, ^12^, ^24^ and the benefits of radiotherapy are more pronounced in patients with advanced disease.^25^ However, radiotherapy complications are not negligible. Acute complications in the early stage of head and neck radiotherapy include acute mucositis, oesophageal dysfunction and dyspepsia, resulting in difficulty in eating and death from malnutrition,^26^ whilst long‐term complications include death from cervical myelopathy, radiation encephalopathy and radiation carcinogenesis.^7^ These complications are also responsible for the reduced survival rate after radiotherapy.^27^

Li et al.^16^ confirmed that ^125^I seed brachytherapy is an attractive option for the slow and continuous release of radiation energy, and its main advantage over external radiotherapy is that it possesses strong conformability and its radioactivity rapidly declines in the surrounding normal tissue, thus providing a biologically higher dose.^28^ ^125^I has a half‐life of approximately 60 days, which allows continuous delivery of radiation to the tumour, whilst ^125^I seed implantation brachytherapy does not interfere with the recovery of damaged nerves.^29^ These properties make it an ideal isotope to be used in the treatment of ACC. It was observed in the present study that surgery combined with ^125^I seed therapy was superior to surgery alone and surgery combined with external radiotherapy, suggesting that ^125^I seed therapy is an effective treatment option for ACC. Patients treated with a combination of surgery and postoperative ^125^I brachytherapy had the highest survival rate of 85.7% (*p* = 0.0059).

Seed therapy had a significant therapeutic effect on the survival of patients with ACC, but further analysis revealed that local recurrence and distant metastasis, which are closely associated with patient survival, were not affected by ^125^I seed therapy. This suggests that ^125^I seed therapy may improve patients' prognosis by inhibiting growth of local recurrent or distant metastatic tumour cells rather than directly suppressing the occurrence of local recurrence and distant metastasis. The findings of the present study revealed that seed therapy is important for the treatment of ACC, but it is necessary to further explore the patient population suitable for ^125^I seed therapy. The patients enrolled in the current study were subjected to dichotomous grouping according to each significant prognostic factor identified through Cox regression analyses in order to further identify the factors that affected the efficacy of ^125^I seed brachytherapy. It was observed that combined surgery and postoperative ^125^I seed brachytherapy achieved a better outcome in patients with T3 and T4 stage tumours (*p* = 0.0049) and those with perineural invasion (*p* = 0.0199) than in those with T1 and T2 stage tumours (*p* = 0.9664) and those without perineural invasion (*p* = 0.2623). This may be explained by the following reasons: (1) due to the large tumour size and invasion of the nerve, it was impossible to delineate a safe boundary for tumour resection; and (2) due to the aggressiveness of ACC, residual, small cancer foci that were invisible to the naked eye persisted, whilst ^125^I seed brachytherapy provided a sustained, localised, high‐dose radiation to inhibit or even kill these residual tumour cells. Given that perineural invasion is an indicator of T4 stage tumours, it is necessary to clarify whether advanced ACC or perineural invasion is an indication for radioactive seed implantation therapy. The results revealed that T4 stage tumours (*p* = 0.0139) were more therapeutically sensitive to combined surgery and postoperative ^125^I seed brachytherapy than were T3 stage tumours (*p* = 0.2382), with perineural invasion serving as a more determining factor of the efficacy of ^125^I seed brachytherapy. That is, the main indication for seed therapy in ACC patients is perineural invasion, regardless of the tumour size.

In the presence of perineural or bone invasion, the tumour‐invaded tissue can be preserved and implanted with ^125^I seeds for internal radiotherapy, which would allow better preservation of the functional appearance and achieve longer survival. The dose of ^125^I seeds was 60–80 Gy for those with negative surgical margins and 80–120 Gy for those with positive surgical margins.^29^, ^30^ Treated patients—regardless of the regimen—achieved better survival than untreated patients. Therefore, a combination of surgery and postoperative ^125^I seed therapy would achieve a better quality of survival and a higher survival rate if the physical condition allows, and those who cannot be operated on can undergo seed therapy^28^ or radiotherapy alone^2^ to inhibit tumour growth and prolong survival. However, seed therapy is not a one–off treatment. If too much soft tissue is removed during surgery, the seeds cannot be fixed around the operated area and should instead be fixed with the help of a prosthetic appliance. The rich masticatory and facial expression muscles of the maxillofacial region may cause loss or migration of seeds during normal facial muscle movements, and the small size of single seeds can lead to serious consequences if they enter the bloodstream and cause pulmonary embolism.^31^

However, this study was subject to a methodological limitation: the patient records may contain insufficient, incomplete or inaccurate data, which may have introduced selection and information bias. Therefore, the survival outcomes and prognostic factors should be accepted with caution. In the future, we will further increase the sample size by recruiting patients from multiple centres and further confirm the conclusions of the present study by improving the follow‐up data. We will also conduct in‐depth exploration of the mechanism of radioactive particle therapy through basic research, provide theoretical guidance for the design and application of particle therapy programs and provide a theoretical basis for the improvement of particle therapy programs.

## 5 CONCLUSION

The present retrospective study of 188 ACC patients revealed that age, regimen, T stage and N stage were significantly associated with prognosis. A combination of surgery and postoperative ^125^I seed implantation therapy significantly improved overall survival, but did not affect local recurrence and distant metastasis. In patients with ACC, perineural invasion was an important indication for radioactive seed implantation.

## AUTHOR CONTRIBUTIONS

**Yutian Cheng:** Conceptualisation, formal analysis, methodology, project administration, writing – original draft, writing – review and editing. **Le Xu:** Formal analysis, methodology, writing – review and editing. **Zhanwei Chen:** Investigation, project administration. **Haiwei Wu:** Investigation, methodology. **Huwei Zou:** Investigation, project administration. **Tianqi Zhang:** Investigation, software. **Guijun Liu:** Data curation, validation. **Zhenxing Liu:** Data curation, validation. **Changwei Yin:** Data curation. **Li Ma:** Data curation. **Shizhou Zhang:** Supervision, writing – review and editing. **Wengang Li:** Supervision, writing – review and editing. **Shengyun Huang:** Conceptualisation, funding acquisition, methodology, supervision, writing – review and editing. **Dongsheng Zhang:** Conceptualisation, funding acquisition, supervision, writing – review and editing.

## CONFLICT OF INTEREST

None.

## ETHICS STATEMENT

This study protocol was approved by the Biomedical Research Ethics Committee of Shandong Provincial Hospital (Approval number: SWYX: No. 2021‐415).

## Data Availability

Research data are not shared due to patient privacy.
